# In this issue: Volume 113, Issue 2, February 2022

**DOI:** 10.1111/cas.14946

**Published:** 2022-02-06

## Biomolecular condensates in cancer biology

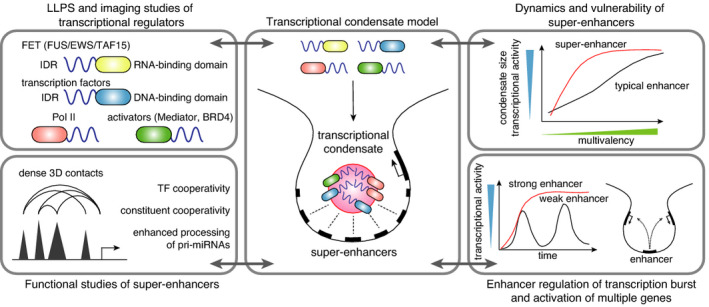

The hallmarks of cancer were initially published in 2000 and laid a basic framework for understanding the differences between normal and malignant cells. How these malignant cells develop the hallmarks despite having nearly identical genomes is not well understood. Studies have suggested that biomolecular condensates play a significant role in gene regulation and cell signaling and could be the mechanism by which cancer cells acquire hallmark traits. In this review, Suzuki and Onimaru summarize the current knowledge of biomolecular condensates and cover examples of condensation alterations in certain cancers. By examining the biophysical dynamics of these condensates, we can gain a better understanding of how cells proceed to malignancies and how these malignancies develop resistance.

https://onlinelibrary.wiley.com/doi/full/10.1111/cas.15232

## Glycosylation of MUC6 by Α1,4‐linked N‐acetylglucosamine enhances suppression of pancreatic cancer malignancy

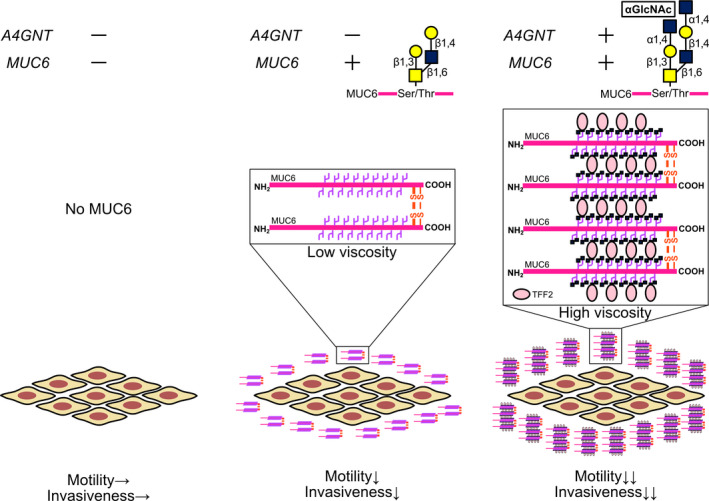

Pancreatic cancer is the 4th leading cause of cancer death worldwide and there have been only marginal improvements in overall survival over the last 30 years. Better biomarkers and therapeutics are sorely needed. Abnormal expression of gastric mucin has been associated with pancreatic tumor progression. Previous studies have shown a decrease in *O*‐linked oligosaccharides with terminal α1,4‐linked *N*‐acetylglucosamine residues (αGlcNAc) and its scaffolding protein MUC6 levels in the early stages of malignant pancreas lesions. In this study, Yuki et al investigated the tumor inhibitory effects of α1,4‐*N*‐acetylglucosaminyltransferase (α4GnT) and MUC6. They found that ectopically expressed MUC6 reduced anchorage dependent cell proliferation, cell motility, and cellular invasiveness. These effects were amplified when MUC6 was glycosylated with αGlcNAc. Analysis of the NCBI Gene Expression Omnibus (GEO) database suggested that higher levels of *A4GNT* and *MUC6* mRNA expression were associated with favorable prognosis, which suggests its potential as a useful biomarker.

https://onlinelibrary.wiley.com/doi/full/10.1111/cas.15209

## Microbial metabolite deoxycholic acid promotes vasculogenic mimicry formation in intestinal carcinogenesis

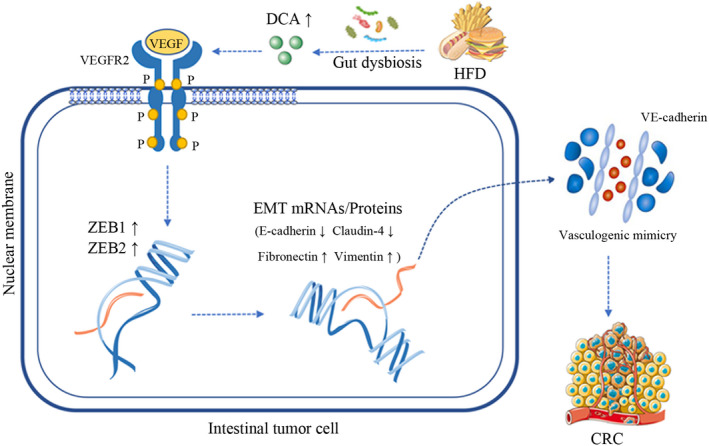

Colorectal cancer (CRC) is the third most commonly diagnosed cancer worldwide. Its incidence is expected to increase significantly in the future and has been associated with the Western diet that trends to be high‐fat diet (HFD). Studies have suggested that HFD results in increased secretion of hepatic primary bile acids, which are further metabolized by gut microbiome into pro‐tumorigenic metabolites like deoxycholic acid (DCA). In this study, Song et al set out to determine whether and how DCA leads to intestinal carcinogenesis. They found that mice fed with HFD had elevated DCA, which promoted cell proliferation and epithelial‐mesenchymal transition (EMT) and increased vasculogenic mimicry (VM) formation, a critical event for the malignant transformation. The group also found that vascular endothelial growth factor receptor 2 (VEGFR2) signaling was vital for these DCA‐induced events. This study improves our understanding of CRC pathogenesis and may provide novel therapeutic targets.

https://onlinelibrary.wiley.com/doi/full/10.1111/cas.15208

